# Clinical implications and immune features of CENPN in breast cancer

**DOI:** 10.1186/s12885-023-11376-2

**Published:** 2023-09-11

**Authors:** Zhengwei Gui, Yao Tian, Tianyao Yu, Shiyang Liu, Chenguang Liu, Lin Zhang

**Affiliations:** Department of Thyroid and Breast Surgery, Tongji Hospital of Tongji Medical College of Huazhong, University of Science and Technology, 1095 Jiefang Avenue, Wuhan City, 430030 Hubei Province China

**Keywords:** CENPN, Breast cancer, Cell proliferation, Immune infiltration, Oncogene

## Abstract

**Background:**

A number of human diseases have been associated with Centromere protein N (CENPN), but its role in breast cancer is unclear.

**Methods:**

A pan-cancer database of Genotype Tissue Expression (GTEx) and the Cancer Genome Atlas (TCGA) were used to examine the expression of CENPN. Using TCGA clinical survival data and breast cancer specimens from our center for validation, the relationship between CENPN expression, breast cancer prognosis, and clinicopathological characteristics of patients was examined. Bioinformatics was utilized to conduct an enrichment study of CENPN. Additionally, the potential of CENPN as a predictive biomarker for immunotherapy success was confirmed by analyzing the co-expression of CENPN with immune-checkpoint related genes, reviewing the TCGA database, and evaluating the correlation between CENPN expression and immune cell infiltration. Using the CCK8 test and colony formation assay, CENPN was evaluated for its ability to inhibit breast cancer cell proliferation. Transwell assays and scratch tests were used to assess the impact of CENPN on breast cancer cell migration.

**Results:**

CENPN is found in a wide range of tumors, including breast cancer. Additional investigation revealed that CENPN was co-expressed with the majority of immune checkpoint-related genes, had the potential to serve as a predictive biomarker for immunotherapy effectiveness, and that high CENPN expression was linked to high Tregs and low CD8 + T cells and NK cells. Breast cancer cells' malignant characteristics, such as migration and cell proliferation, were inhibited by CENPN knockdown.

**Conclusions:**

According to our findings, CENPN may be an oncogene in breast cancer, as well as a new therapeutic target for immune checkpoint inhibitors.

**Supplementary Information:**

The online version contains supplementary material available at 10.1186/s12885-023-11376-2.

## Background

An increasing amount of individuals are at risk for developing breast cancer, one of the most commonly diagnosed cancers in women today. Combining surgery, chemotherapy, endocrine therapy, radiotherapy and targeted therapy are the current treatments for it. Breast cancer treatment still faces tremendous challenges because of tumor heterogeneity and drug resistance. There is currently no established treatment standard for this pathological stage, particularly for triple-negative breast cancer. As a result, finding new therapeutic targets and causal genes through screening, identification, and validation is crucial for the treatment of breast cancer.

A class of protein-coding genes called centromere proteins play an important role in the assembly of kinetochores during mitosis and chromosome segregation. Mitogens recruit the kinetochore protein complex, which aids in the directional transfer of replicating pairs of chromosomes to the meiotic spindle structure, where the replicated genome is distributed from the mother cell to the daughter cell. Several cancers have been linked to members of the CENPs family. CENPA, a prognostic biomarker, is linked to metastasis, recurrence, and advanced disease status. The expression of centromere protein F (CENPF) is upregulated in pancreatic cancer [1]、esophageal squamous cell carcinoma [2] and breast cancer [3]and is involved in cell proliferation, migration, and epithelial mesenchymal transition in pancreatic cancer, and high expression of CENPF is associated with poor prognosis in pancreatic cancer patients. The expression of centromere protein H (CENPH) was upregulated in cervical cancer [4]、breast cancer [5]、and gastric cancer [6], promoting the proliferation of gastric cancer cells and tongue cancer cells, and it could be used as an independent prognostic biomarker for cervical cancer and breast cancer.

CENPN is a constituent of the Cenplatin protein family, which collectively constitute the Constitutive Cenplatin-Associated Network (CCAN). The CCAN is categorized into five sub-complexes based on their cellular biological functions: the CENP-C complex, the CENP-LN complex, the CENP-HIKM complex, the CENP-OPQRU complex, and the CENP-TWSX complex. Conversely, CENPLN serves as the central nexus that connects the entire CCAN network. It interacts with CENPA and facilitates the recruitment of the remaining four protein complexes. The N-terminus of CENP-N establishes a direct interaction with a distinct loop within CENP-A, thereby facilitating the recruitment of CENP-N to the mitophagosome during interphase [7, 8]. Conversely, the C-terminus of CENP-N directly interacts with CENP-L and forms a binding association with CENP-C and CENP-HIKM through this interaction [9, 10]. The phosphorylation of the CENPLN complex occurs upon initiation of mitosis, leading to the disruption of the interaction between a specific subset of CENPN and CENPL molecules, resulting in their dissociation from the mitosome. Following phosphorylation during mitosis, the dephosphorylation of CENPL and CENPN is necessary during the S phase in order for them to relocalize to the mitosome.

Past studies have shown that CENPN is overexpressed in lung adenocarcinoma and promotes its tumor progression [11],CENPN also promotes malignant biological behavior in nasopharyngeal carcinoma cells by enhancing aerobic glycolysis [12]. However, the role of CENPN in breast cancer has not been revealed.

## Materials and methods

### Data collection and processing

All statistical analyses and visualizations were performed using R (https://www.r-project.org) and Graphpad Prism version 8.0. Bioinformatics analyses were conducted using the TCGA and GEO databases. There were 179 paracancerous tissues and 1065 breast cancer tissues altogether. Data on the survival curve were retrieved from the KM plotter website (http://www.kmplot.com) [13].

### Correlation and enrichment analyses

In order to screen the coding genes among them and arrange them in descending order by Pearson correlation coefficient, select the top 50 genes for heat maps, and extract the top 300 genes for GSEA enrichment analysis, the data in TCGA-BRCA were analyzed using the stat package of R software (version 3.6.3). To conduct the GSEA enrichment analysis, the top 300 genes were taken in. For GO/KEGG pathway enrichment analysis, the 261 genes having absolute values of Foldchange larger than 1.5 were chosen.

### Cell culture and treatment

Shanghai Institute of Cell Biology (Shanghai, China) provided MCF7, MDA-MB-231, MDA-MB-468, and SKBR3 human breast cancer cell lines. At 37 °C, cell lines were grown in a ThermoFisher incubator containing 5% CO2. By using STR to identify all purchased cell lines and comparing them to reliable databases.

### Pathological sample collection

From September 2020 to February 2022, we collected 76 breast cancer specimens from Tongji Hospital, with 52 cases of paraffin-embedded tissues, 21 of which included paired specimens of cancer and paracancerous tissues. In 24 cases, fresh frozen tissues were collected, including cancer and paracancer paired specimens. This study was approved by Ethics Committee of Tongji Hospital in accordance with the Helsinki Declaration (approval number TJIRB20221218).

### Pathological sample processing

We fixed tumor and paracancerous tissues in 10% formalin, paraffin-embedded them, serially sectioned them into 5 mm sections, dewaxed them, rehydrated them, and microwaved them. The specimens were incubated at 1 degree Celsius with CENPN (AFFINITY, df2315) antibody diluted at 1:100. After 30 min at room temperature, secondary antibodies were stained with DAB substrate and re-stained with hematoxylin. ImageJ and AI software were used to perform quantitative immunohistochemistry analysis.

### RNA extraction and qRT-PCR

CENPN and GAPDH primers were obtained from DynaScience Biotechnology. TRIzol reagent (Invitrogen, USA) was used for total RNA isolation. Primers sequences (5'-3') were as follows: CENPN: forward: ACAAACCTACCTACGTGGTGT, reverse: CCAGAAGCGGTGTATTGCG. GAPDH forward—GGAGCGAGATCCCTCCAAAAT, reverse—GGCTGTTGTCATACTTCTCATGG. There were forty PCR cycles at 95 °C for 5 min (95 °C for 5 s, 60 °C for 30 s). Relative expression levels were normalized to the internal control and calculated according to the 2-ΔΔCT method.

### CCK8 assay

Each experimental group digested and resuspended in full culture media. Cell proliferation was measured at 1d, 2d, 3d, and 4d using the CCK-8 (Invitrogen, USA). In 450 nm, optical densities were measured using an enzyme marker (Molecular Devices, Rockford, IL).

### Colony-formation assay

Inoculating 3,000 breast cancer cells onto six-well plates, they were then cultivated for 14 days. Crystal violet (Beyotime, China) was used to dye the cell colonies after they had been fixed by 4% polyacetal soaking for 10 min. Images and counts of the cell colonies were taken.

### Transwell assay

20,000 cells are seeded in the transwell upper chamber (Corning, USA). After 24 h at 37 °C, the cells were wiped from the top surface. The bottom surface of the chambers was fixed with 4% paraformaldehyde, then stained with crystal violet for 10 min. And then, migrating cells were imaged and counted.

### Scratch test

IBIDI two-well culture inserts were incubated for 24 h in 24-well plates with healthy 231 and MCF-7 breast cancer cells in the log phase of growth. On the pristine table, forceps were used to delicately remove the culture implants. Each well received 1 mL of low-serum media, and at 0 and 1 day after the inserts were withdrawn, a rate of cell migration was observed under a light microscope.

### Immune cell infiltration

Immune cell infiltration in BC was analyzed victimisation the GSVA package of R [14]. The results were based on ssGSEA. 24 immune cells were classified and Markers were referenced to previous studies [15]. The degrees of immune cell infiltration in TCGA-BRCA samples were assessed based on the median CENPN expression (high vs. low). The Cancer Genome Atlas (TCGA) dataset contained RNAseq data (level3) and associated clinical data for 1101 breast tumors. Using the TIDE algorithm (Tumor Immune Dysfunction and Exclusion), the probable immunotherapeutic response was predicted.

### Flow cytometry analysis

The cells were collected, subsequently washed twice with phosphate-buffered saline (PBS), and incubated with V-FITC and PI for a duration of 15 min. The analysis of apoptosis was then conducted using flow cytometry, following the instructions provided by the manufacturer. Flow cytometry data analysis was performed using FlowJo software (Treestar, USA).

### Immunofluorescence microscopy

The cells were treated with a 4% paraformaldehyde solution for a duration of 15 min. Subsequently, actin and nuclei were stained using a rhodamine ghost pen cyclic peptide with a concentration of 2.5 units/ml and DAPI, respectively. Finally, the stained cells were examined using fluorescence microscopy.

### ELISA

Culture medium was obtained from MDA-MB-231 and MCF-7 cells, followed by the implementation of an Enzyme-Linked Immunosorbent Assay (ELISA) to ascertain the concentration of individual cytokines. The absorbance at 450 nm was quantified using an enzyme marker, subsequently determined through the utilization of a standard curve, and subsequently expressed as picograms per milliliter (pg/ml).

## Results

### Patient characteristics

The cohort consisted of 1065 BC patients with clinical information and RNA sequencing data, 110 of whom were matched to adjacent normal tissue samples from the TCGA. Using the GTEx database, gene expression data for normal breast tissues were added. Table S1 shows the clinicopathological characteristics of these patients.

### CENPN expression analysis

**Fig. 1:**
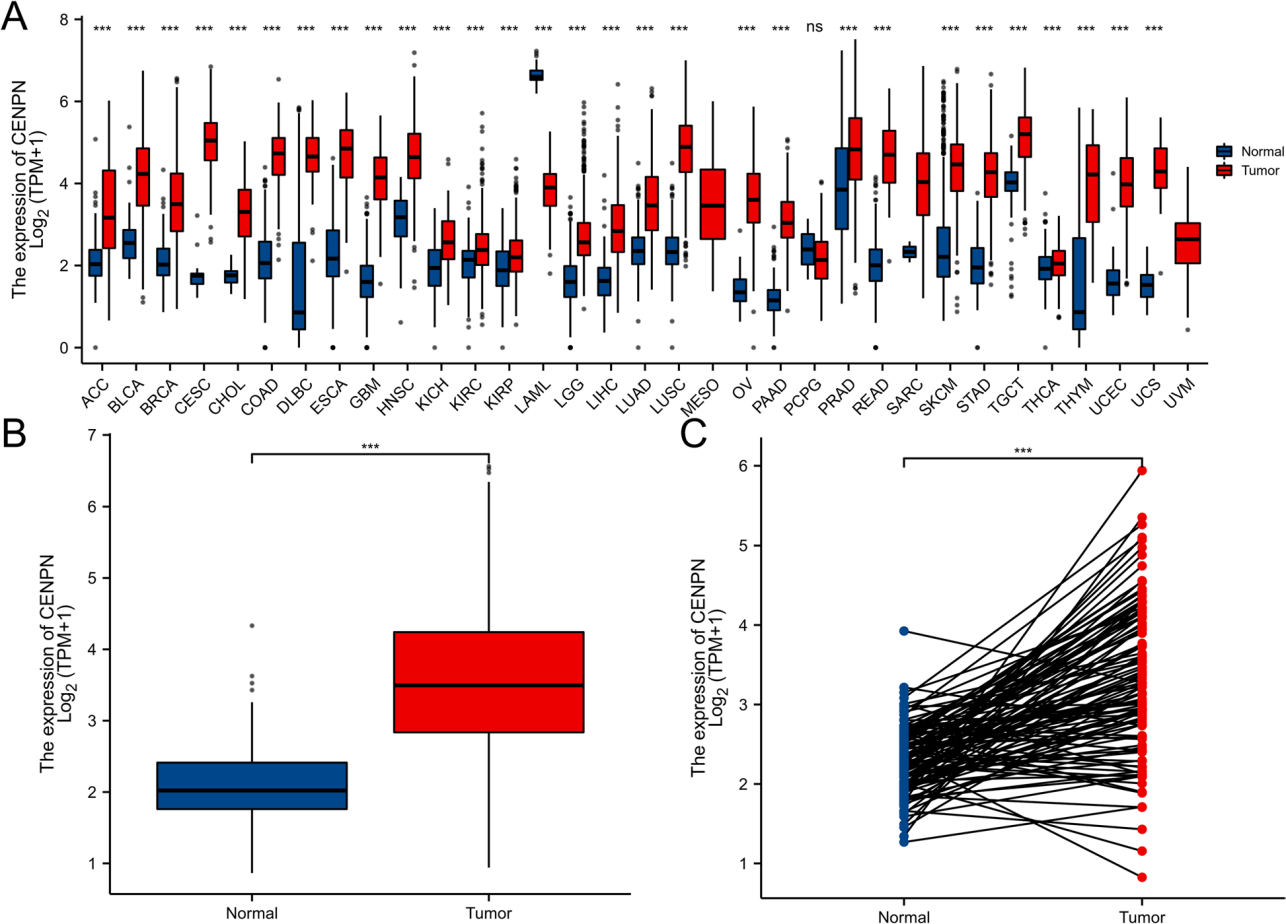
The expression difference of CENPN in cancer tissue and normal tissue. Expression of CENPN in pan-cancer and adjacent normal tissues in TCGA and GTEx databases. **B** Expression of CENPN in unpaired breast cancer samples in TCGA-BRCA database. **C** Expression of CENPN in paired breast cancer samples in TCGA-BRCA database. Data were shown as mean ± SD. *p < 0.05, **p < 0.01, ***p < 0.001

In the TCGA and GTEx pancancer databases, CENPN expression was higher in 28 tumors than in normal tissues. (Fig. 1A).

Unpaired (Fig. 1B) and paired (Fig. 1C) TCGA breast cancer samples had significantly high expression levels.

### The relationship between CENPN expression and the prognosis of breast cancer patients

**Fig. 2:**
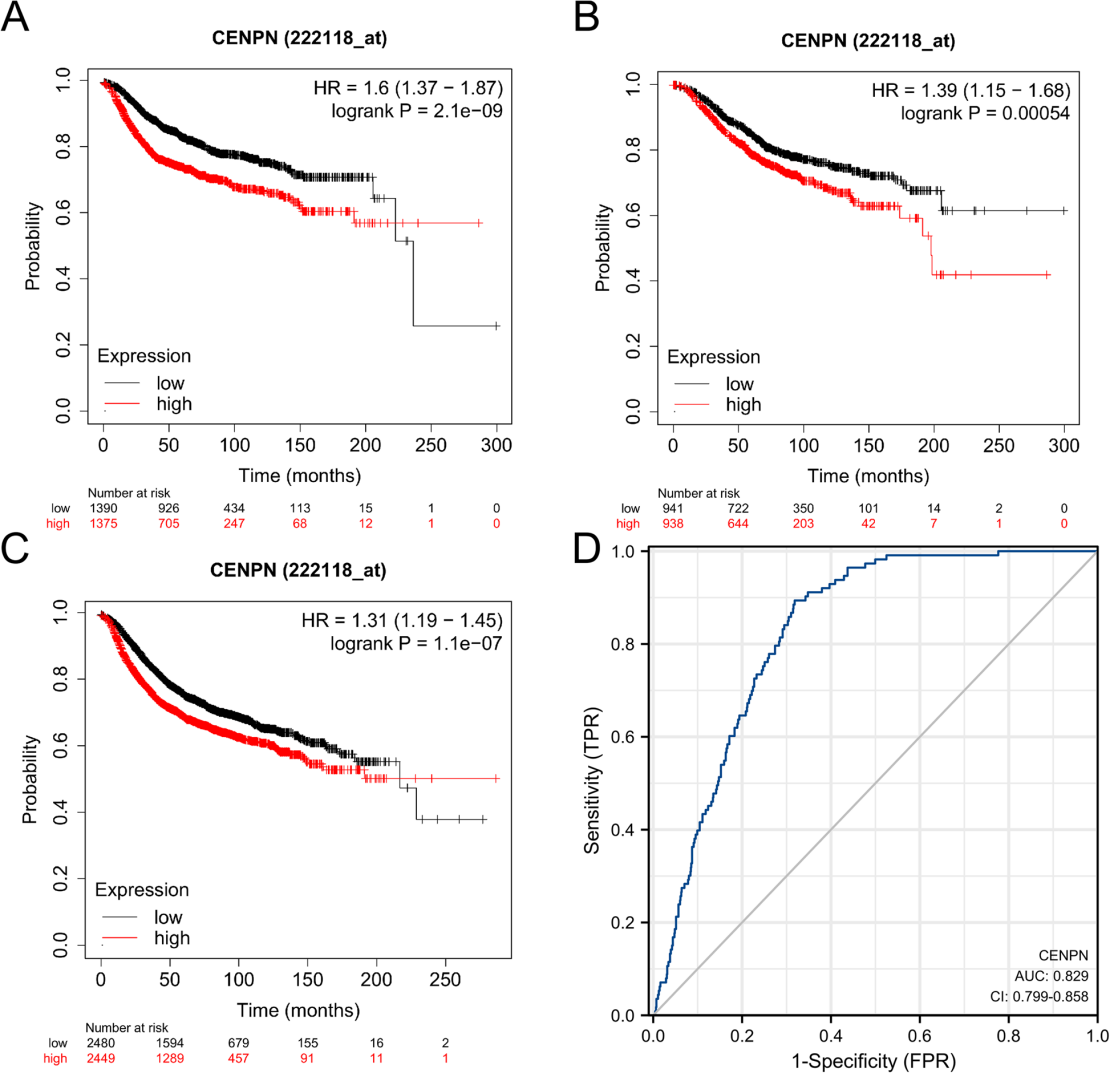
Expression of CENPN and prognosis of breast cancer patients. **A** OS of breast cancer patients based on CENPN expression level. **B** RFS of breast cancer patients based on CENPN expression level. **C** DMFS of breast cancer patients based on CENPN expression level. **D** ROC curve of CENPN

As a means of assessing the utility of CENPN expression in predicting cancer patient prognosis, we examined the relationship between CENPN expression and DMFS (Fig. 2A), OS (Fig. 2B), and RFS (Fig. 2C) in the TCGA cohort. The findings revealed that patients with high CENPN expression in breast cancer had a poor prognosis. (DMFS: HR = 1.6, *P* = 2.1e-09; OS: HR = 1.39, *P* = 0.00054; RFS: HR = 1.31, *P* = 1.1e-07) The ROC curves (Fig. 2D) of the TCGA-GTEx-BRCA database for the diagnosis of breast invasive cancer were plotted (AUC = 0.829, CI:0.799–0.858).

### Clinicopathologic variables associated with CENPN expression

**Fig. 3:**
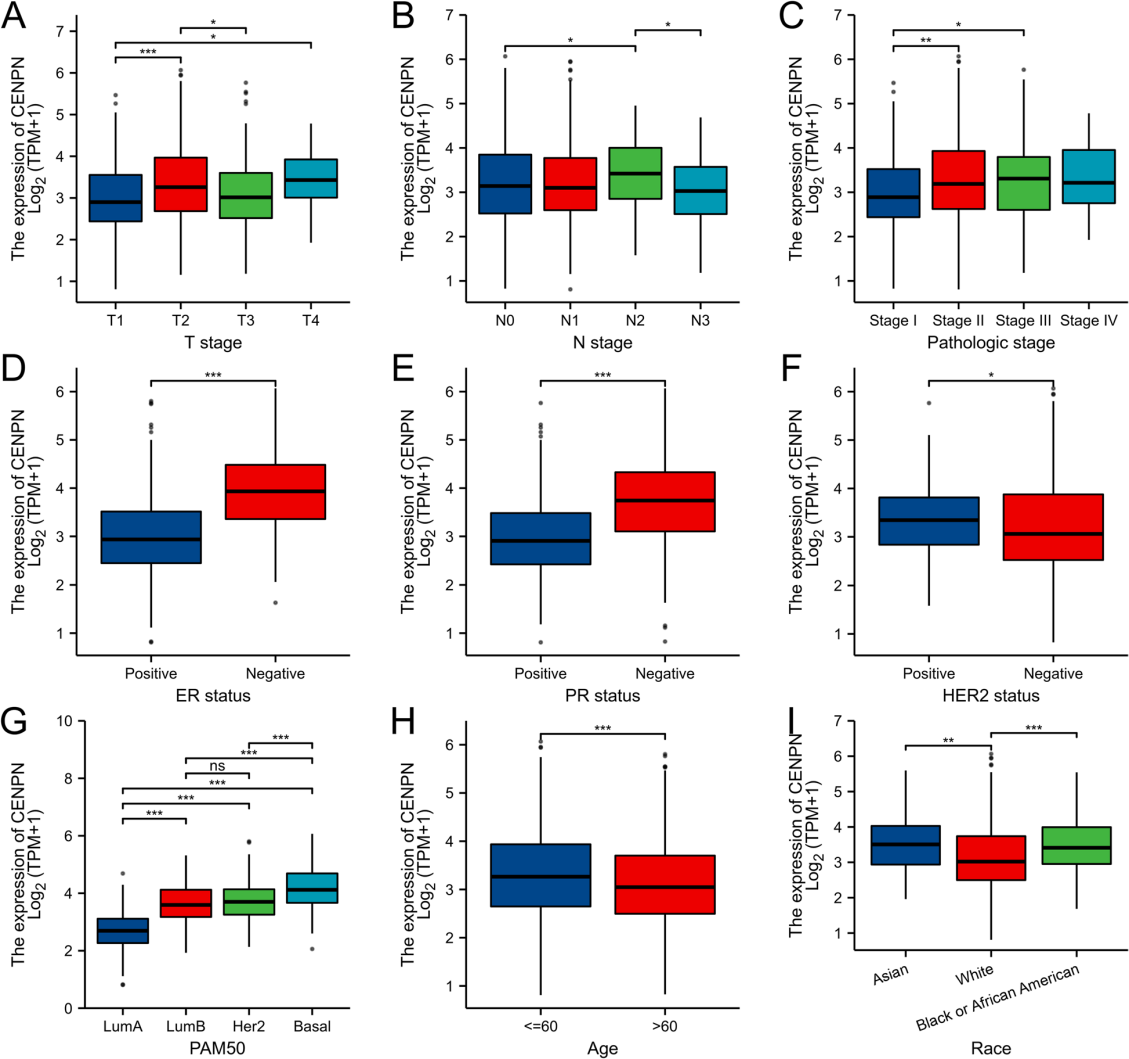
Relationship between CENPN expression and clinicopathologic features of breast cancer patients in TCGA. Data are shown for (**A**) T stage; (**B**) N stage; (**C**) Pathologic stage; (**D**) ER status; (**E**) PR status; (**F**) HER-2 stage; (**G**) PAM50; (**H**) Age; (**I**) Race; *p < 0.05, **p < 0.01, ***p < 0.001. LumA, Luminal A; LumB, Luminal B; ER, estrogen receptor; PR, progesterone receptor; HER2, human epidermal growth factor receptor 2

**Fig. 4:**
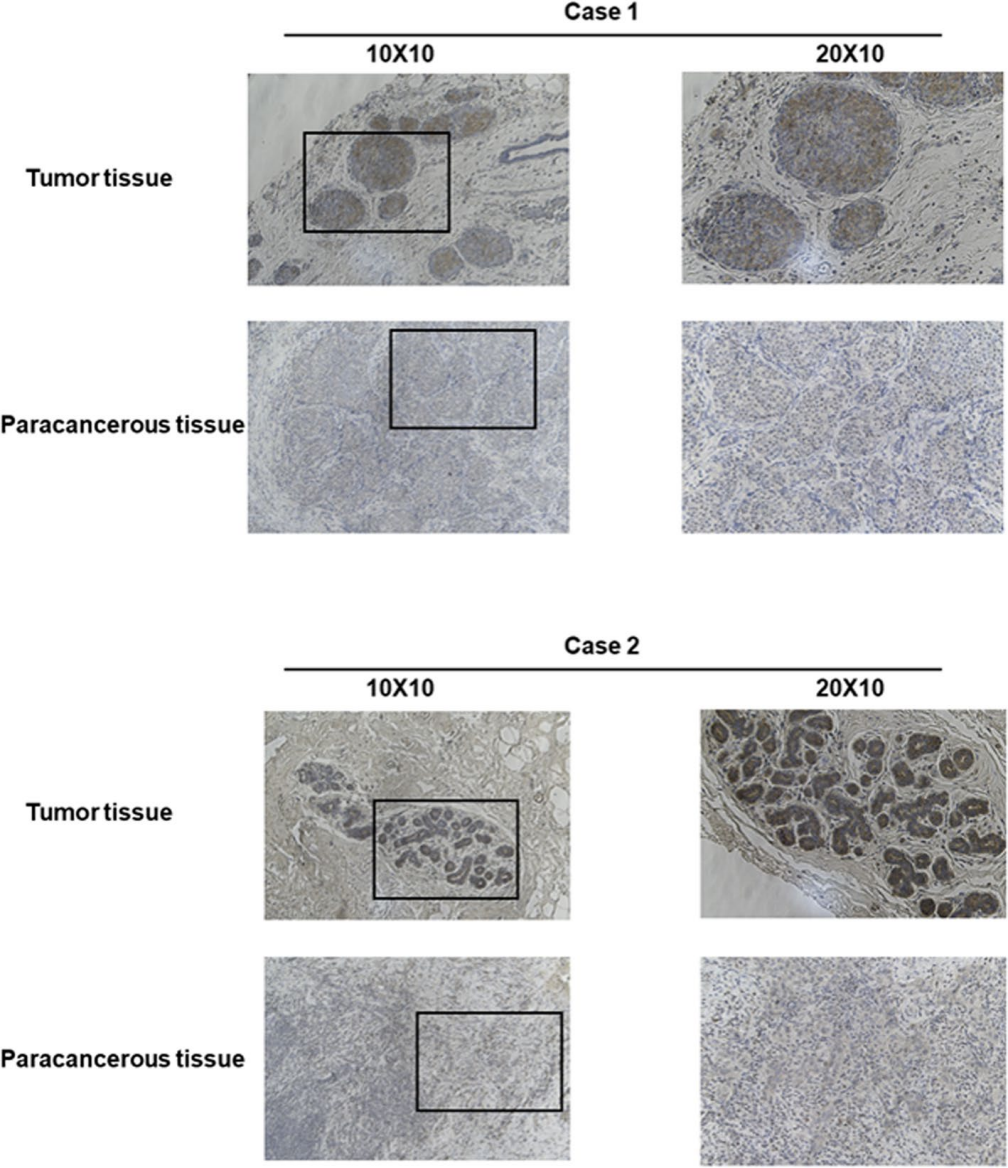
Representative images of CENPN expression in breast cancer tissues and their matched paracancerous tissues. Original magnifications 100 × and 200 × (inset panels)

**Fig. 5:**
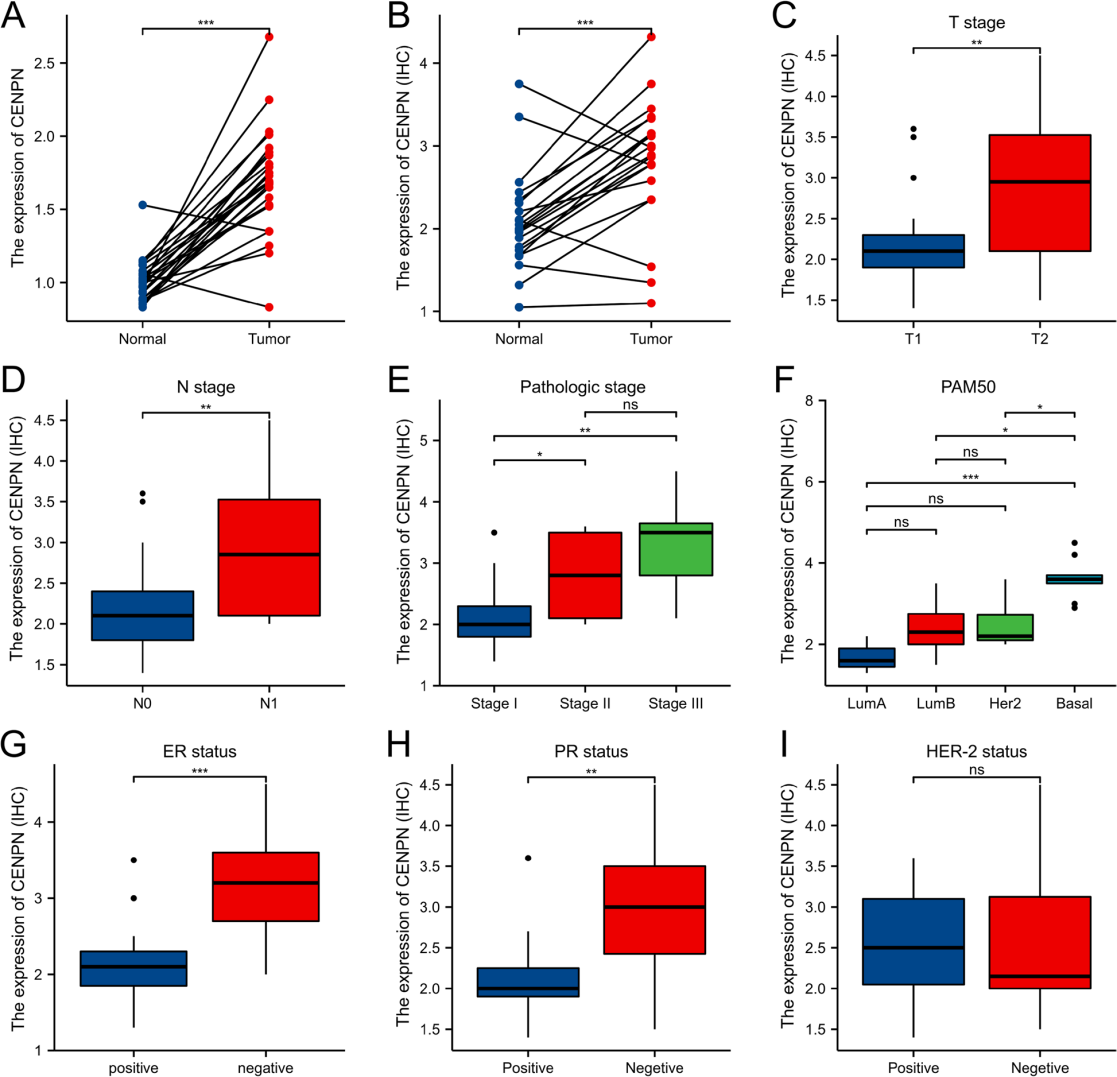
Expression and the relationship between CENPN and breast cancer clinicopathologic features in our center. **A** mRNA levels of CENPN in 24 pairs of fresh frozen specimens (**B**) Protein levels of CENPN in 21 pairs of paraffin sections (**C**) T stage; (**D**) N stage; (**E**) Pathologic stage; (**F**) PAM50; (**G**) ER status; (**H**) PR status; (**I**) HER-2 status; *p < 0.05, **p < 0.01, ***p < 0.001

In Table S 1 and Fig. 3, CENPN expression was associated with T stage, ER status, PR status, and histological type; PAM50; Race and Age were related. The overexpression of CENPN in breast cancer was verified at the transcriptional (Fig. 5A) and translational (Fig. 5B) levels in breast cancer specimens collected at our center, respectively. A typical immunohistochemical picture is shown in Fig. 4. Immunohistochemistry scores were utilized to statistically analyze the clinicopathological data of the patients, which revealed that CENPN overexpression was associated with higher T-stage, N-stage, and pathological stage (Fig. 5C, D and E) and was highly expressed in ER (Fig. 5H), PR (Fig. 5G), and triple-negative breast cancer patients than in other types of breast cancer (Fig. 5F), and these results were generally consistent with those obtained from bioinformatic analysis. However, immunohistochemical results of our specimens showed that the expression of CENPN was not related to HER-2 status (Fig. 5I).

### Correlation and enrichment analyses

**Fig. 6:**
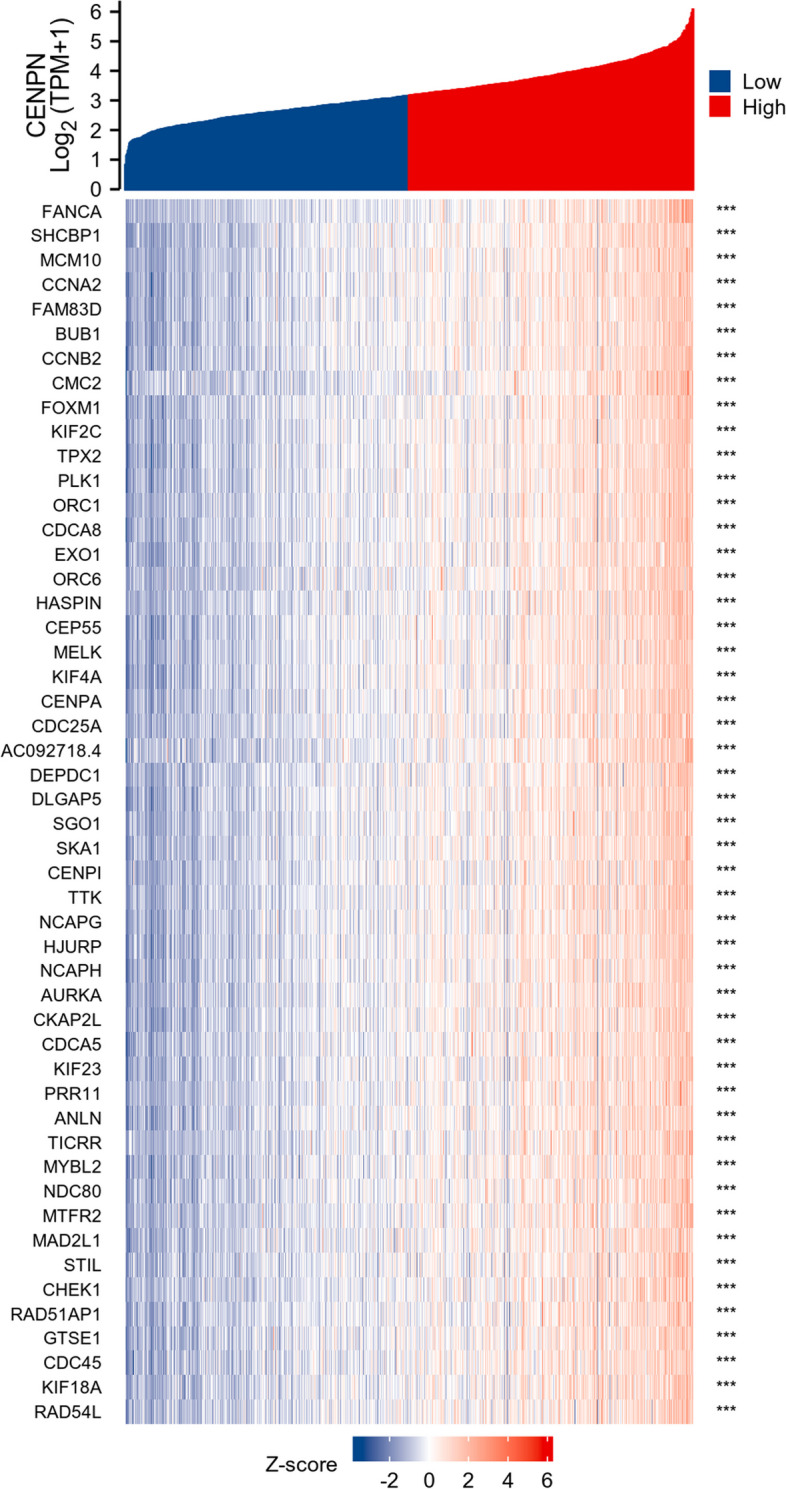
The 50 co-expressed genes with the highest positive correlation of CENPN

**Fig. 7:**
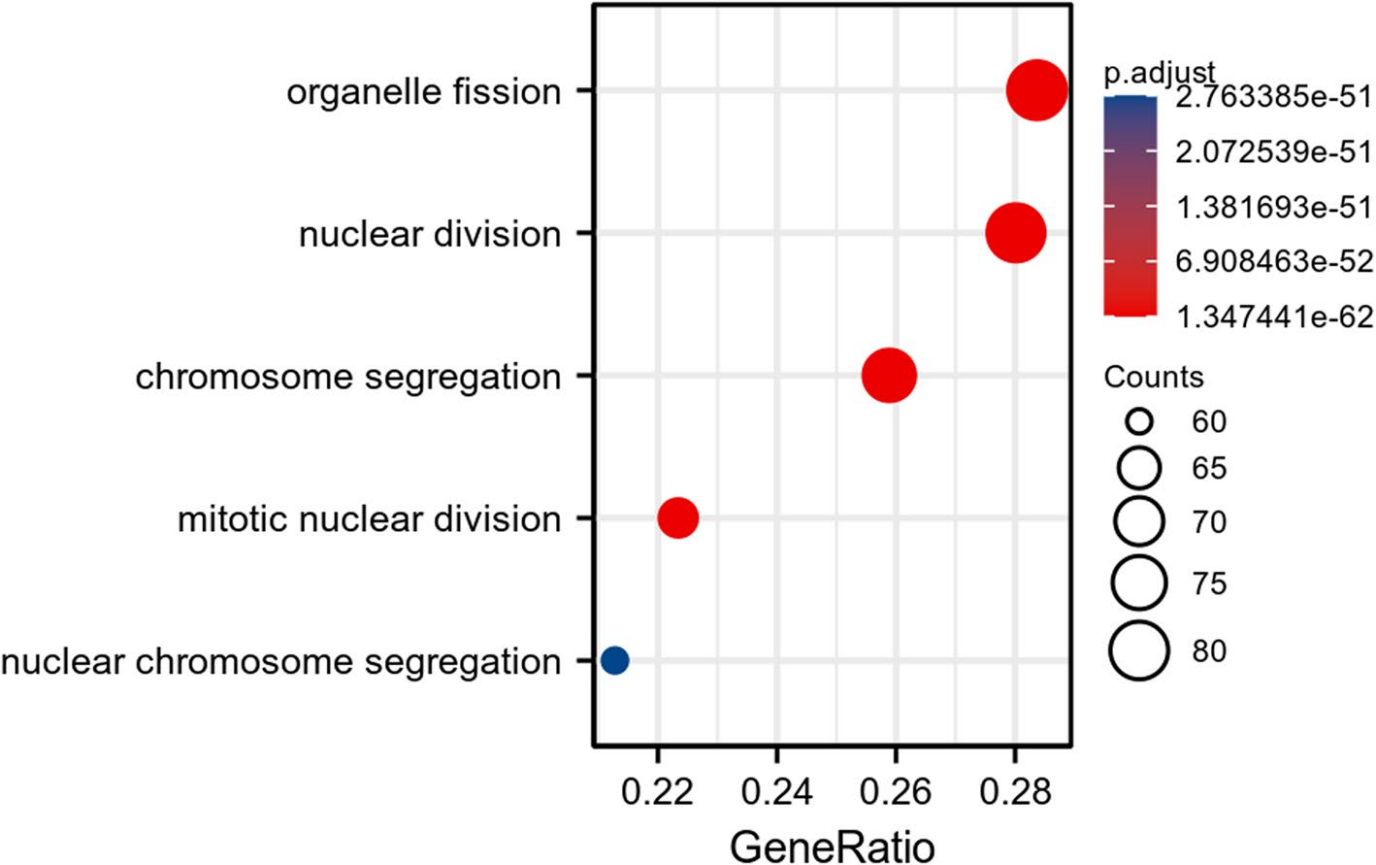
GSEA pathway enrichment analysis of CENPN

**Fig. 8:**
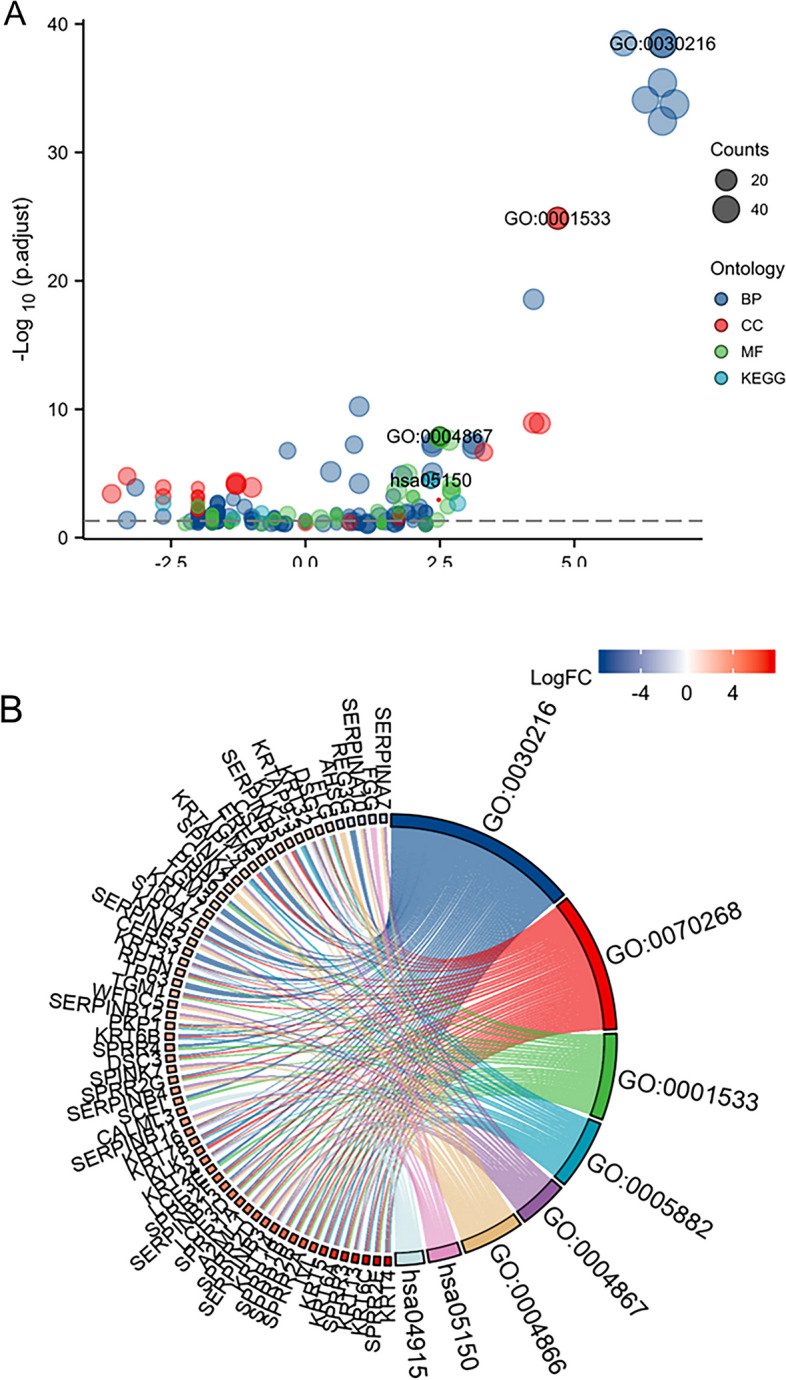
GO and KEGG pathway enrichment of CENPN. **A** GO/KEGG enrichment bubble diagram. **B** Enrichment string diagrams of GO/KEGG. BP: cornification(z-score = 4.000), keratinization(z-score = 3.638) CC: intermediate filament cytoskeleton(z-score = 3.207), intermediate filament(z-score = 2.886). MF: neurotransmitter transporter activity(z-score = 2.449), neurotransmitter:sodium symporter activity(z-score = 2.000)

The data in TCGA-BRCA were analyzed using the stat package of R software to obtain a list of molecular correlations co-expressed with CENPN, and the coding genes were filtered out and arranged in descending order by Pearson correlation coefficients, and the first 50 genes were selected to draw a heat map (Fig. 6). The top 300 genes were extracted for GSEA enrichment analysis (Fig. 7) and GO/KEGG pathway analysis (Fig. 8), which showed that CENPN was mainly associated with cell proliferation-related pathways.

### Expression of CENPN and immune cell infiltration

**Fig. 9:**
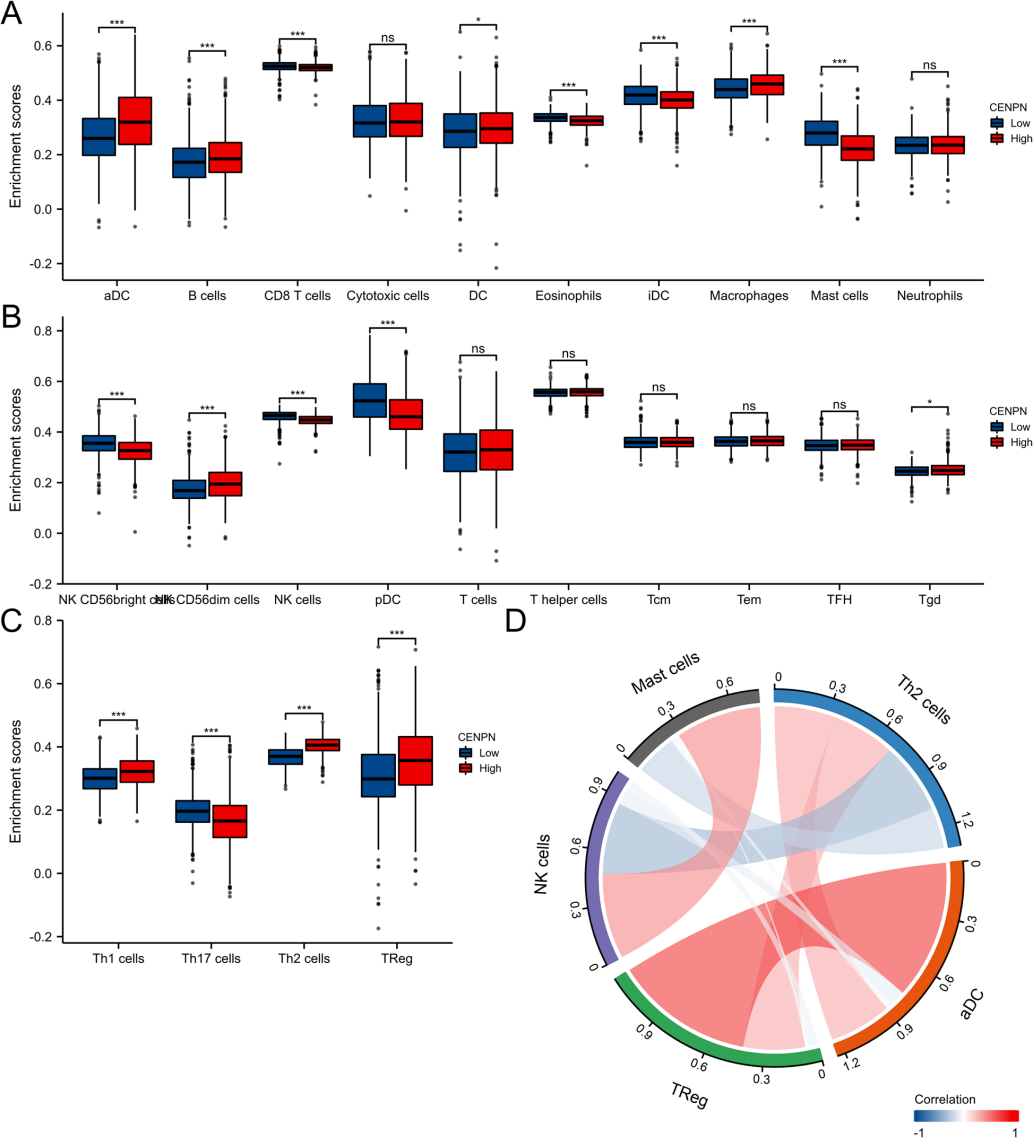
Analysis of CENPN and immune cell infiltration groups. **A**-**C** Grouping of immune cells based on CENPN expression levels. **D** Correlation of 5 immune cells

**Fig. 10:**
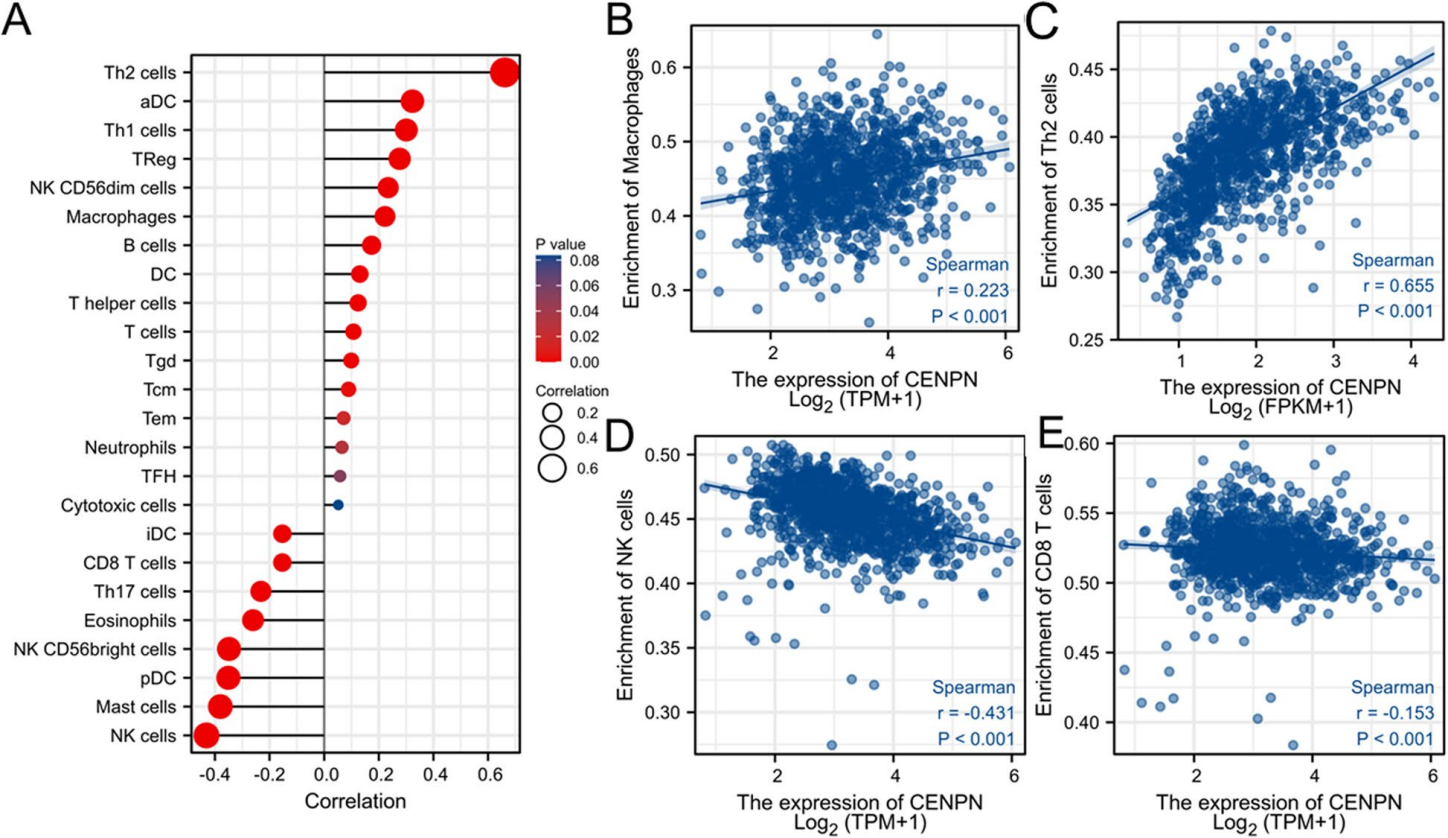
Associated between CENPN with immune cell infiltration. **A** Correlation between the expression level of CENPN and various immune cell infiltration. **B** Correlation between CENPN expression and macrophages. **C** Correlation between CENPN expression and Th2 cells. **D** Correlation between CENPN expression and NK cells. **E** correlation between CENPN expression and CD8 + T cells

**Fig. 11:**
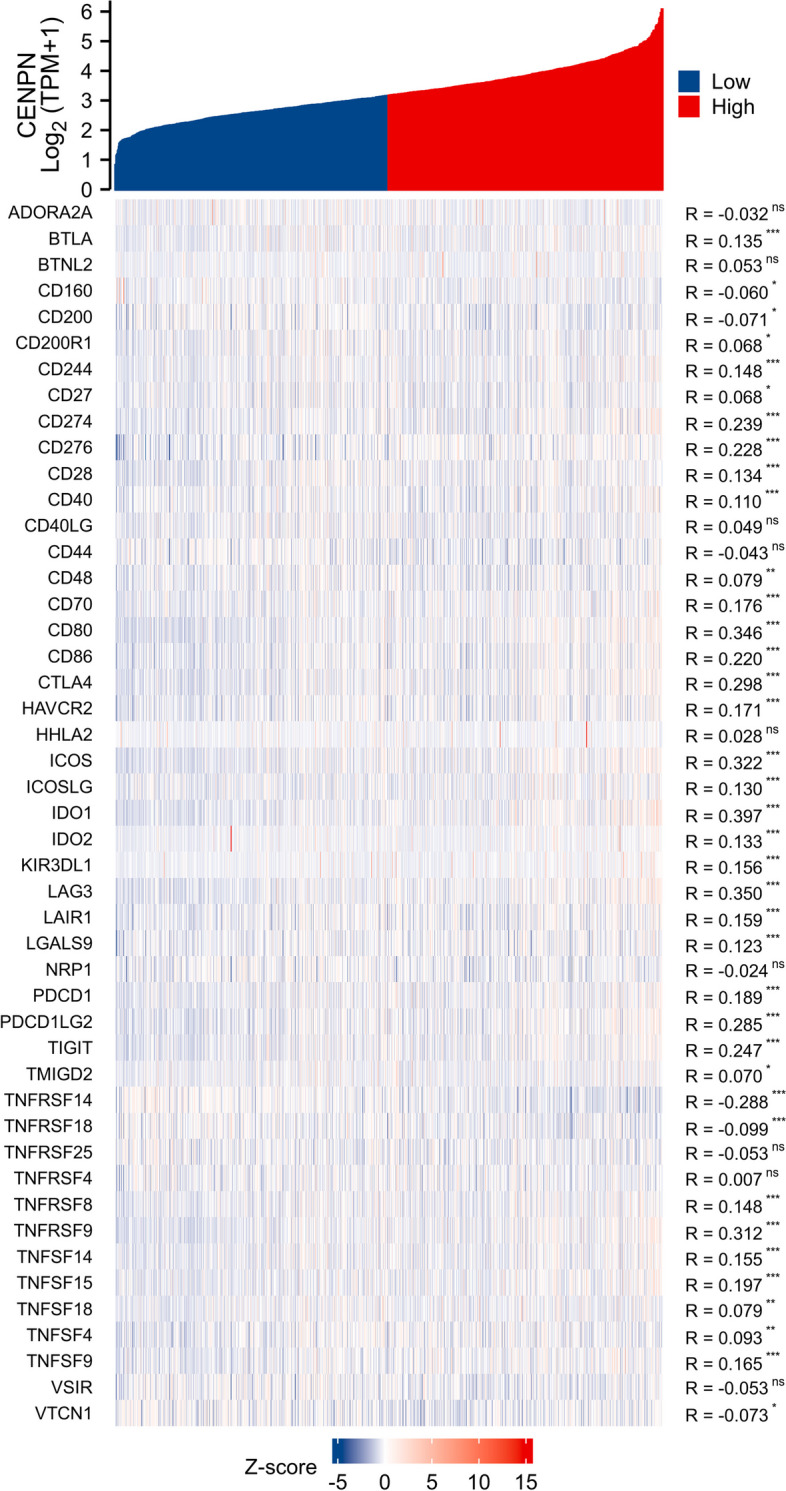
Co-expression of CENPN and immune checkpoint related genes

**Fig. 12:**
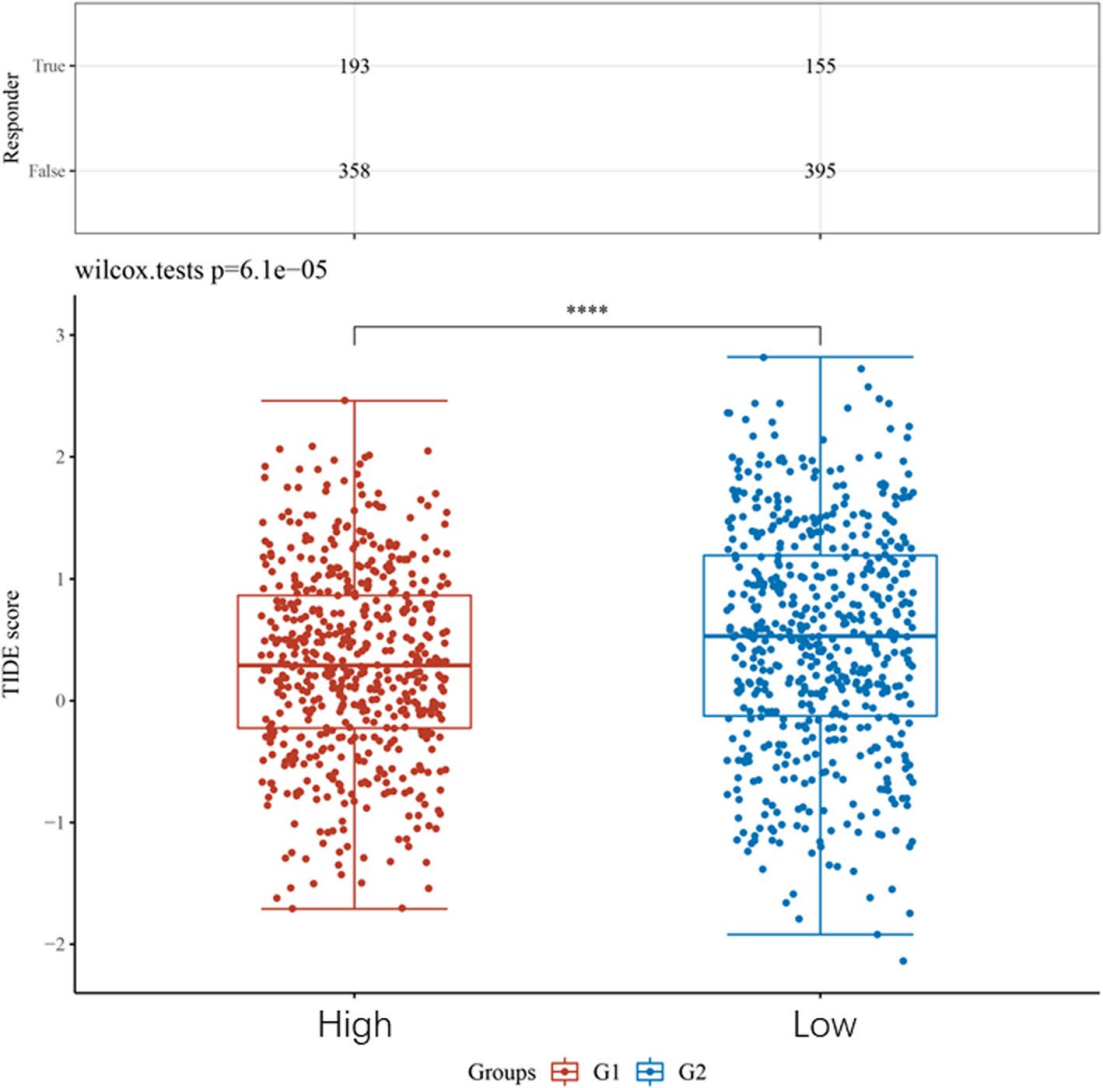
TIDE based on the expression level of CENPN

Then, using the TCGA database, we examined the immune cell infiltration scores of breast cancer patients (Fig. 9). According to Fig. 10, high CENPN expression encourages the intra-tumor accumulation of Tregs and Th2, suppressed CD8 + T cells, and NK cells. The CENPN expression was related to high Tregs, Th2, and low CD8 + T cells. According to these findings, breast cancer tumor immunosuppression is strongly correlated with increased CENPN expression. In order to examine the relationship between CENPN and ICs, the researchers chose 47 genes linked to ICs in previous studies [16], and investigated CENPN's co-expression with the aforementioned genes (Fig. 11). These genes co-expressed with CENPN in close to 80% of cases. Breast tumors with CENPN overexpression appeared to be more amenable to immunotherapy, according to TIDE study [17, 18] (Fig. 12).

### A CENPN knockdown inhibited breast cancer cells' malignant behavior

**Fig. 13:**
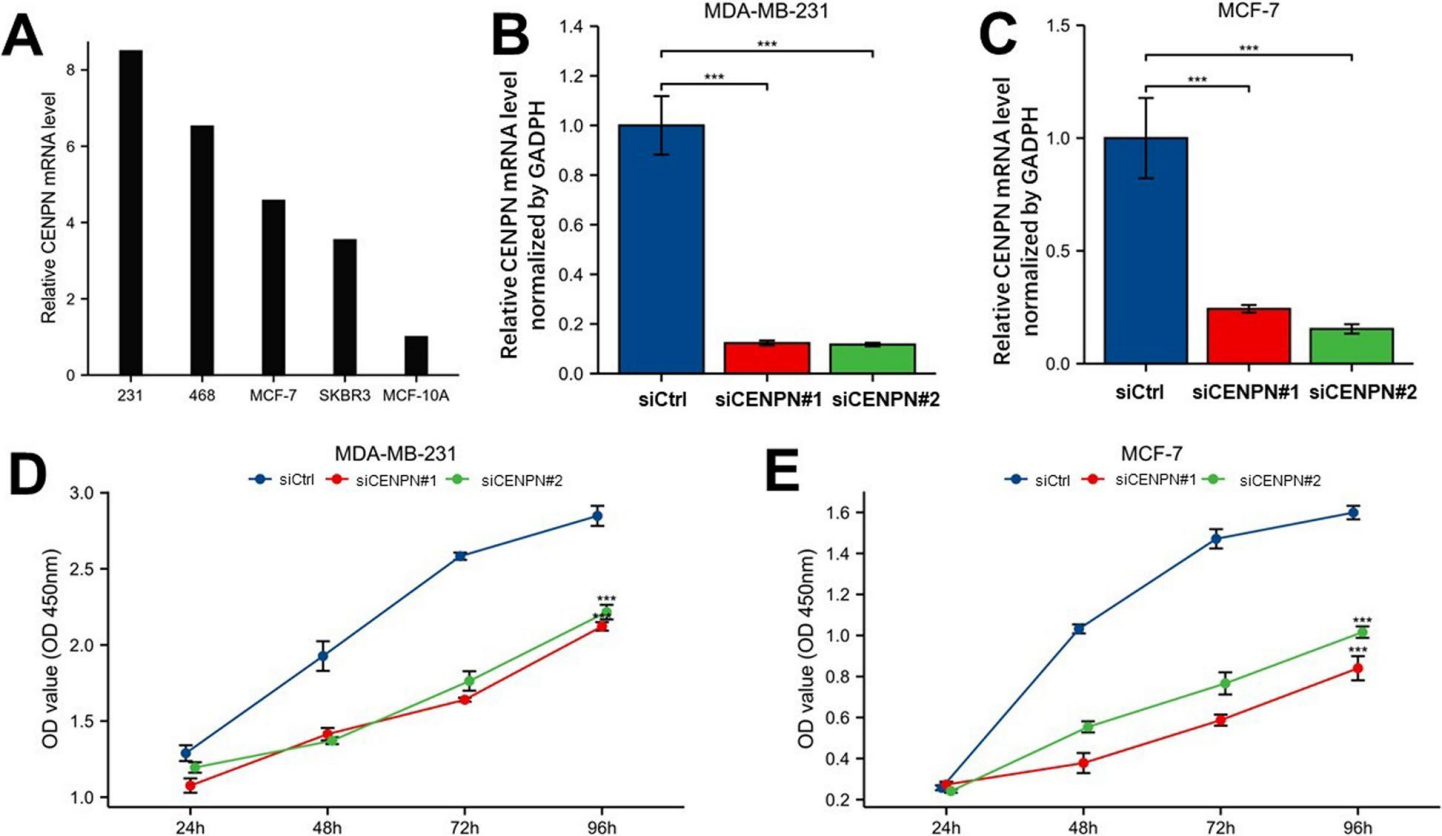
Expression and knockdown of CENPN in various cell lines and CCK8 cell proliferation experiment. **A** CENPN expression in MDA-MB-231, MDA-MB-468, MCF7, SKBRE3, and MCF10A cell lines. **B** CENPN knockdown efficiency of two siRNA in MDA-MB-231 cell lines. **C** knockdown of two siRNA in MCF7 cell lines Efficiency of CENPN. **D**-**E** Cell proliferation in two siRNA knockout groups and control groups in MDA-MB-231 and MCF7 cell lines

**Fig. 14:**
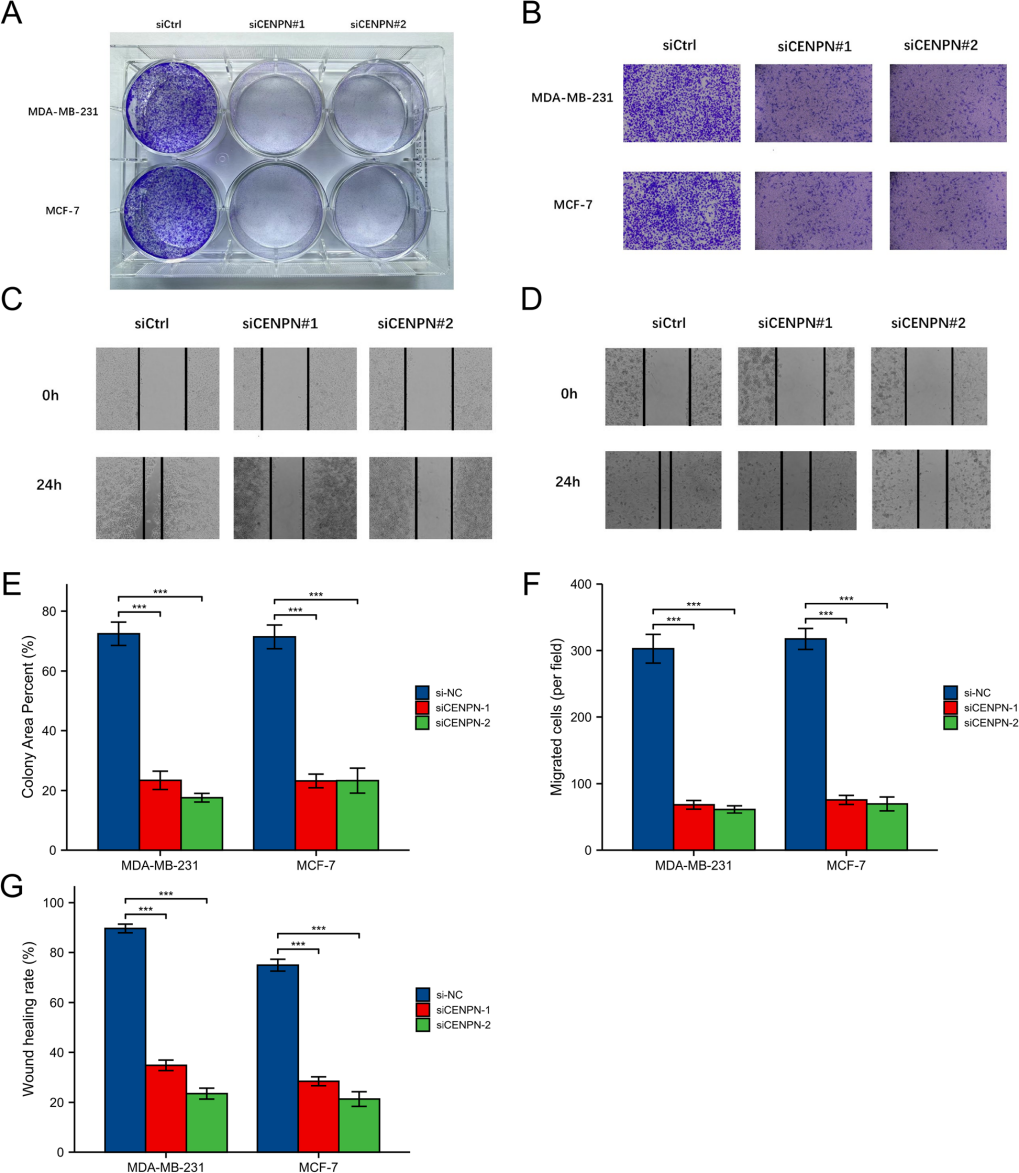
Clone formation experiment, Transwell experiment and scratch experiment. Clone formation of control group and two siRNA knockout groups in MDA-MB-231 and MCF7 cell lines. **B** Transwell images of control group and two siRNA knockout groups in MDA-MB-231 and MCF7 cell lines. **C**–**D** Scratch test images of control group and two siRNA knockout groups in MDA-MB-231 and MCF7 cell lines. **E** Quantitative analysis of clone formation experiment. **F** Quantitative analysis of transwell experiment. **G** Quantitative analysis of scratch experiment. All assays were independently repeated at least three times. Data are presented as the mean ± SD. * *p* < 0.05, **p < 0.01, ***p < 0.001, **** *p* < 0.0001

Furthermore, we check the expression of CENPN in other breast cancer cell lines and discovered that, in contrast to MCF-10A cells, which represent normal breast cells, CENPN was highly expressed in 468, 231, MCF-7, and SKBR3 cells. (Fig. 13A) The expression of CENPN in 231 and MCF-7 cells was silenced using two siRNAs targeting CENPN, and we investigated the biological effects on these two cell lines. (Fig. 13B, C) Then, we measured cell proliferation using CCK8 assays. The outcomes showed that CENPN knockdown drastically reduced the rate of proliferation of both cell lines (Fig. 13D, E). By using a colony formation assay, CENPN's detrimental impact on BC cell proliferation was further demonstrated (Fig. 14A). Transwell test and scratch assay results also showed that CENPN silencing dramatically reduced breast cancer cells' capacity to migrate. (Fig. 14B–D) Transwell, scratch, and clone generation assays were all subjected to quantitative analysis (Fig. 14E–G). In conclusion, CENPN encourages biological processes that lead to cancer, such as the migration and proliferation of BC cells.

### Analysis of the mode of cell death

**Fig. 15:**
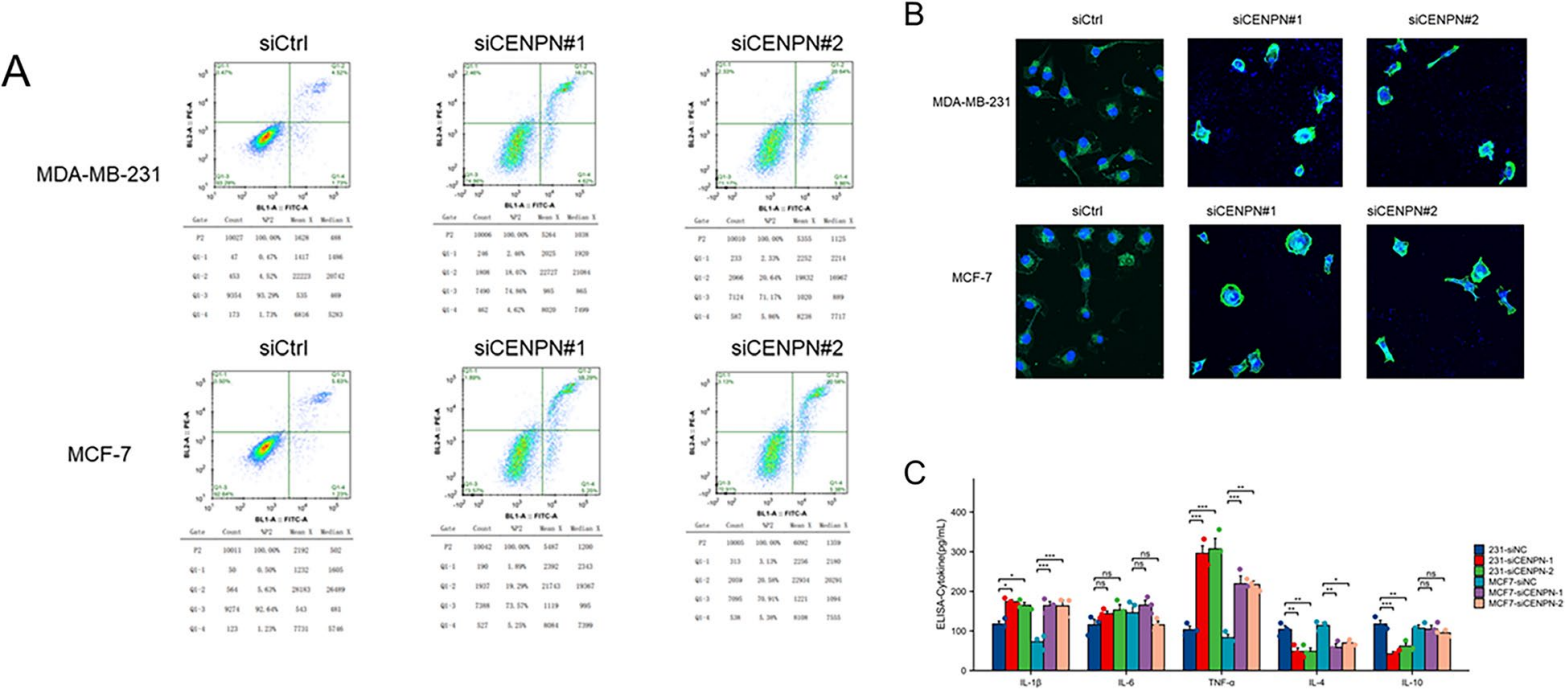
Analysis of the mode of cell death. **A** Apoptosis assay by flow cytometric analysis. **B** Confocal fluorescence micrographs. **C** The cytokine profile was measured by ELISA

To further investigate the cell death pattern of breast cancer cells following CENPN knockdown, flow cytometry was employed by the researchers to detect apoptosis (Fig. 15A). The obtained results demonstrated a significant increase in early apoptosis, as well as middle and late apoptosis, in both MDA-MB-231 cells and MCF-7 cells after CENPN knockdown. Additionally, confocal imaging provided a more visually comprehensive representation of the morphological changes observed in apoptotic cells. (Fig. 15B) Cytokine levels in the supernatants of cell cultures were detected using the ELISA assay. The CENPN knockdown cell group exhibited up-regulation of classical pro-inflammatory cytokines, namely IL-1β and TNF-α, while down-regulation of anti-inflammatory cytokines, specifically IL-4 and IL-10, was observed (Fig. 15C).

## Discussion

The 16 types of centromere proteins, including CENPC, CENPI, CENPK, CENPH, CENPN, CENPM, CENPL, CENPO, CENPP, CENPQ, CENPT, CENPS, CENPR, CENPX, CENPW, and CENPU are called constitutive The CCAN (constitutive centromere-associated network) [19]. The CCAN is roughly divided into five subcomplexes: the CENPC complex, the CENPLN complex, the CENPHIKM complex, the CENPOPQRU complex, and the CENPTWSX complex. Although the CCAN protein complex is localized to the mitophagus throughout the cell cycle, it is not a static, unchanging structure. Instead, it is constantly reorganized during different intervals of the cell cycle through interactions between the various subcomplexes. This means that the assembly of CCAN is dependent on the interactions of these protein complexes. 16 contacts for ongoing reconfiguration at various cell cycle stages [20].

Among CCAN's subcomplexes, the CENPLN complex plays an important role in the formation of the kinetochore. By direct interactions with CENPL, the C-terminus of CENPN connects to the CENPC and CENPHIKM complexes. CENPN and CENPL efficiently connect the interface between mitophilic chromatin and the formed CCAN through this interaction [7]. During interphase, CENPN is recruited to the centromere protein by interacting directly with a unique circuit within CENPA [8, 21]. Bioinformatic analysis and cellular tests used in our work have demonstrated that CENPN is overexpressed in a range of malignancies, including breast cancer, and that this overexpression has a significant impact on patient prognosis. It primarily controls cell proliferation pathways and is linked to a number of biological characteristics that are malignant, including the proliferation, migration, and invasion of BC cells.

In spite of the immune system's ability to clear tumor cells through immune circulation, the incidence of malignant tumors continues to rise each year because tumor cells can avoid immune surveillance and clearance by altering the immunological milieu of the tumor to a state of immunosuppression [22]. Cancer immune microenvironments comprise tumor cells, immune cells, cytokines, etc. These components interact to cause the tumor immune microenvironment to exhibit two different trends of anti-tumor or pro-tumor [23]. Different immune cells in the tumor immune microenvironment have antagonistic effects on each other. Among them, immune cells with antitumor effects include: cytotoxic T lymphocytes [24], helper T cell subpopulation Th1, M1-type macrophages [25], NK cells and antigen-presenting dendritic cells. In contrast, immune cells with pro-tumor effects include: Tregs [26], M2-type macrophages [27], myeloid-derived suppressor cells, N2-polarized neutrophils, and NKT 2 cells. The primary research directions in tumor immunotherapy today are the stimulation of anti-tumor immune cells and the inhibition of pro-tumor cells in tumor tissues. Our research found that CENPN overexpression lowered the aggregation of antitumor immune cells (CD8 + T cells and NKs) and boosted the infiltration of immunosuppressive cells (Tregs and Th2 cells) within breast cancer, which may be the mechanism of its encouragement of breast tumor development.

Both studies, KEYNOTE-522 [28] and Impassion 130 [29] advanced treatment for TNBC using ICIs from advanced to early stages. The frequency of pCR in patients was related to PD-L1 expression in cancers and tumor-infiltrating lymphocytes, according to the KEYNOTE-173 [30] study, in which pabrolizumab was used in conjunction with neoadjuvant chemotherapy in patients with high-risk early-stage TNBC. However, overall, pabrolizumab in combination with neoadjuvant chemotherapy demonstrated manageable side effects. The findings demonstrated a relationship between tumor expression and tumor-infiltrating lymphocytes and the patient's pCR rate. As a result, the U.S. FDA approved pablizumab for use as ongoing monotherapy adjuvant therapy following surgery and in conjunction with neoadjuvant chemotherapy for high-risk early-stage TNBC. Screening the target population for appropriate immunotherapy has become essential for the accurate and effective therapeutic delivery of immunotherapy as a result of the growth of the indications for ICIs. Our research demonstrates that the majority of immune checkpoint-related genes co-express with CENPN, indicating that CENPN may interact with several immune checkpoint-related pathways. In TIDE, two distinct tumor escape mechanisms are analyzed, as malfunction of tumor-infiltrating CTL and immunosuppression of CTL, using gene expression markers. A high TIDE score was linked to poor ICB efficacy and short survival following ICB treatment. In TIDE, immunotherapy was more effective in treating breast tumors with high CENPN expression, raising the possibility that it could be used as a biomarker for predicting treatment effectiveness of immune checkpoint inhibitors and serve as a potential novel immunotherapy target.

Our study's limitation is that more long-term follow-up data are required for further validation of the utility of CENPN for anticipating immunotherapy in breast cancer.

## Conclusions

According to our findings, CENPN may be an oncogene in breast cancer, as well as a new therapeutic target for immune checkpoint inhibitors.

### Supplementary Information

**Additional file 1.**  Clinicopathologic variables associated with CENPN expression.  

## Data Availability

The datasets used and/or analyzed during the present study are available from the corresponding author on reasonable request.
