# Supplementary material for: Clinical implications and immune features of CENPN in breast cancer
Source: BMC Cancer. 2023 Sep 11;23:851. doi: 10.1186/s12885-023-11376-2 (PMC10496242; doi:10.1186/s12885-023-11376-2)
Supplement: Supplementary file 1 — Additional file 1. Clinicopathologic variables associated with CENPN expression. [file 12885_2023_11376_MOESM1_ESM.docx]

# Clinicopathologic variables associated with CENPN expression

| Characteristic | Low expression of CENPN | High expression of CENPN | p |
| --- | --- | --- | --- |
| n | 541 | 542 |  |
| T stage, n (%) |  |  | < 0.001 |
| T1 | 166 (15.4%) | 111 (10.3%) |  |
| T2 | 286 (26.5%) | 343 (31.8%) |  |
| T3 | 75 (6.9%) | 64 (5.9%) |  |
| T4 | 13 (1.2%) | 22 (2%) |  |
| N stage, n (%) |  |  | 0.020 |
| N0 | 263 (24.7%) | 251 (23.6%) |  |
| N1 | 184 (17.3%) | 174 (16.4%) |  |
| N2 | 42 (3.9%) | 74 (7%) |  |
| N3 | 41 (3.9%) | 35 (3.3%) |  |
| M stage, n (%) |  |  | 0.588 |
| M0 | 439 (47.6%) | 463 (50.2%) |  |
| M1 | 8 (0.9%) | 12 (1.3%) |  |
| Pathologic stage, n (%) |  |  | 0.029 |
| Stage I | 108 (10.2%) | 73 (6.9%) |  |
| Stage II | 299 (28.2%) | 320 (30.2%) |  |
| Stage III | 112 (10.6%) | 130 (12.3%) |  |
| Stage IV | 8 (0.8%) | 10 (0.9%) |  |
| Race, n (%) |  |  | < 0.001 |
| Asian | 17 (1.7%) | 43 (4.3%) |  |
| Black or African American | 65 (6.5%) | 116 (11.7%) |  |
| White | 420 (42.3%) | 333 (33.5%) |  |
| Age, n (%) |  |  | 0.005 |
| <=60 | 277 (25.6%) | 324 (29.9%) |  |
| >60 | 264 (24.4%) | 218 (20.1%) |  |
| Histological type, n (%) |  |  | < 0.001 |
| Infiltrating Ductal Carcinoma | 316 (32.3%) | 456 (46.7%) |  |
| Infiltrating Lobular Carcinoma | 170 (17.4%) | 35 (3.6%) |  |
| ER status, n (%) |  |  | < 0.001 |
| Negative | 40 (3.9%) | 200 (19.3%) |  |
| Indeterminate | 0 (0%) | 2 (0.2%) |  |
| Positive | 480 (46.4%) | 313 (30.2%) |  |
| PR status, n (%) |  |  | < 0.001 |
| Negative | 91 (8.8%) | 251 (24.3%) |  |
| Indeterminate | 2 (0.2%) | 2 (0.2%) |  |
| Positive | 427 (41.3%) | 261 (25.2%) |  |
| HER2 status, n (%) |  |  | 0.002 |
| Negative | 302 (41.5%) | 256 (35.2%) |  |
| Indeterminate | 6 (0.8%) | 6 (0.8%) |  |
| Positive | 60 (8.3%) | 97 (13.3%) |  |
| PAM50, n (%) |  |  | < 0.001 |
| Normal | 30 (2.8%) | 10 (0.9%) |  |
| LumA | 429 (39.6%) | 133 (12.3%) |  |
| LumB | 49 (4.5%) | 155 (14.3%) |  |
| Her2 | 15 (1.4%) | 67 (6.2%) |  |
| Basal | 18 (1.7%) | 177 (16.3%) |  |
| Menopause status, n (%) |  |  | 0.106 |
| Pre | 101 (10.4%) | 128 (13.2%) |  |
| Peri | 19 (2%) | 21 (2.2%) |  |
| Post | 366 (37.7%) | 337 (34.7%) |  |
| Anatomic neoplasm subdivisions, n (%) |  |  | 0.006 |
| Left | 258 (23.8%) | 305 (28.2%) |  |
| Right | 283 (26.1%) | 237 (21.9%) |  |
| radiation_therapy, n (%) |  |  | 0.153 |
| No | 235 (23.8%) | 199 (20.2%) |  |
| Yes | 273 (27.7%) | 280 (28.4%) |  |
| Age, median (IQR) | 60 (50, 68) | 56 (47, 66) | < 0.001 |
